# Clinical and Laboratory Differences between Lymphocyte- and Neutrophil-Predominant Pleural Tuberculosis

**DOI:** 10.1371/journal.pone.0165428

**Published:** 2016-10-27

**Authors:** Hayoung Choi, Hae Ri Chon, Kang Kim, Sukyeon Kim, Ki-Jong Oh, Suk Hyeon Jeong, Woo Jin Jung, Beomsu Shin, Byung Woo Jhun, Hyun Lee, Hye Yun Park, Won-Jung Koh

**Affiliations:** Division of Pulmonary and Critical Care Medicine, Department of Medicine, Samsung Medical Center, Sungkyunkwan University School of Medicine, Seoul, South Korea; Rutgers Biomedical and Health Sciences, UNITED STATES

## Abstract

Pleural tuberculosis (TB), a form of extrapulmonary TB, can be difficult to diagnose. High numbers of lymphocytes in pleural fluid have been considered part of the diagnostic criteria for pleural TB; however, in many cases, neutrophils rather than lymphocytes are the predominant cell type in pleural effusions, making diagnosis more complicated. Additionally, there is limited information on the clinical and laboratory characteristics of neutrophil-predominant pleural effusions caused by *Mycobacterium tuberculosis* (MTB). To investigate clinical and laboratory differences between lymphocyte- and neutrophil-predominant pleural TB, we retrospectively analyzed 200 patients with the two types of pleural TB. Of these patients, 9.5% had neutrophil-predominant pleural TB. Patients with lymphocyte-predominant and neutrophil-predominant pleural TB showed similar clinical signs and symptoms. However, neutrophil-predominant pleural TB was associated with significantly higher inflammatory serum markers, such as white blood cell count (*P* = 0.001) and C-reactive protein (*P* = 0.001). Moreover, MTB was more frequently detected in the pleural fluid from patients in the neutrophil-predominant group than the lymphocyte-predominant group, with the former group exhibiting significantly higher rates of positive results for acid-fast bacilli in sputum (36.8 versus 9.4%, *P* = 0.003), diagnostic yield of MTB culture (78.9% versus 22.7%, *P* < 0.001) and MTB detected by polymerase chain reaction (31.6% versus 5.0%, *P* = 0.001). Four of seven patients with repeated pleural fluid analyses revealed persistent neutrophil-predominant features, which does not support the traditional viewpoint that neutrophil-predominant pleural TB is a temporary form that rapidly develops into lymphocyte-predominant pleural TB. In conclusion, neutrophil-predominant pleural TB showed a more intense inflammatory response and a higher positive rate in microbiological testing compared to lymphocyte-predominant pleural TB. Pleural TB should be considered in neutrophil-predominant pleural effusions, and microbiological tests are warranted.

## Introduction

Tuberculosis (TB) is a major global health burden, developing in an estimated 9.6 million patients and contributing to 1.5 million deaths annually [1]. In areas with high TB prevalence, pleural TB is the most common form of extrapulmonary TB and the main cause of pleural effusion [2–4]. The gold standard of pleural TB diagnosis requires detection of *Mycobacterium tuberculosis* (MTB) in pleural fluid or histological demonstration of a caseous granuloma in the pleura [5–8]. However, staining for acid-fast bacilli (AFB) and MTB culturing of pleural fluid have low sensitivity due to the paucibacillary nature of the disease, and pleural biopsy is an invasive procedure associated with high complication rates [5–8]. Therefore, pleural fluid analysis is essential for diagnosing pleural TB.

Previously, lymphocyte-predominant exudates with high adenosine deaminase (ADA) have been classified as pleural TB [5–8]. Nonetheless, the diagnosis of pleural TB using pleural fluid analysis is still challenging. While previous studies have shown that lymphocytes constitute up to 90% of total cells in pleural fluid with pleural TB [9, 10], recent retrospective studies have reported that the level of lymphocytes in pleural fluid decreased in patients who were diagnosed with pleural TB [11, 12]. Moreover, neutrophil-predominant pleural fluid is often encountered in pleural TB. However, there are limited data on the clinical characteristics of neutrophil-predominant pleural TB compared with lymphocyte-predominant pleural TB [11]. Thus, we aimed to investigate differences in the clinical and laboratory characteristics of lymphocyte- and neutrophil-predominant pleural TB through analyses of patient serum, sputum, and pleural fluid.

## Materials and Methods

### Study population

This study included consecutive, adult patients (age ≥18 years) with newly diagnosed pleural TB at Samsung Medical Center (a 1,979-bed referral hospital in Seoul, Korea) between January 2009 and May 2014. All included patients underwent thoracentesis at least once during diagnostic workup. Pleural TB was diagnosed as indicated in the section “Diagnostic criteria”. Patients who were diagnosed or who had received anti-TB treatment at other hospitals were not included. The Institutional Review Board (IRB) of Samsung Medical Center approved this study, and permission to review and publish information obtained from patient records was obtained (IRB No. 2016-03-040). Informed consent was waived for the use of patient medical data, and patient information was anonymized and de-identified prior to analysis.

### Diagnostic criteria

**Fig 1:**
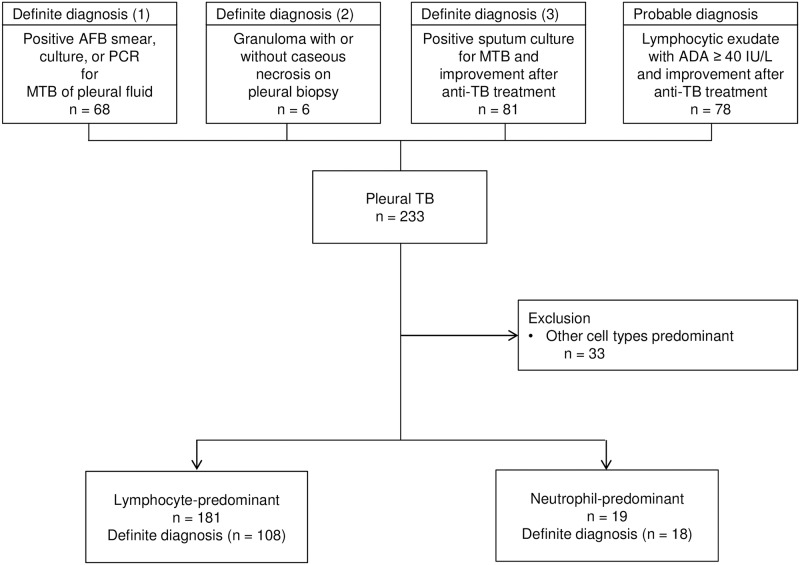
Groupings of study population according to diagnostic criteria and cell type predominance. TB, tuberculosis; AFB, acid-fast bacilli; PCR, polymerase chain reaction; MTB, *Mycobacterium tuberculosis*; ADA, adenosine deaminase.

Patients were diagnosed with definite pleural TB based on (1) a positive AFB smear, growth of MTB in culture, or detection of MTB by polymerase chain reaction (PCR), using pleural fluid as the source specimen; (2) a pleural biopsy revealing granuloma, with or without caseous necrosis, in the absence of other causes of granulomatous lung disease; or (3) positive sputum culture for TB with improvement of the pleural effusion after anti-TB treatment. Probable pleural TB is defined as a lymphocytic exudate with ADA ≥ 40 IU/L, in the absence of malignant evidence, along with improvement after anti-TB treatment (Fig 1) [12].

Lymphocyte and neutrophil predominance were defined, respectively, as when the percentage of lymphocytes or neutrophils was higher than 50% of the total fluid leukocyte count. Basophils, monocytes, and eosinophils were grouped as “other” cell types.

Lung parenchymal lesions were defined as pulmonary findings, such as micronodules, consolidation, and cavitation, that were compatible with pulmonary TB on initial chest computed tomography (CT) [13–15]. For patients without an available chest CT, patchy or nodular shadows and cavitation that were observed on the initial chest X-ray and that improved after anti-TB treatment were considered to be parenchymal lesions [16].

### Microbiological tests

AFB staining was performed with an auramine-rhodamine fluorescent stain, followed by confirmation with Ziehl-Neelsen staining. Staining results were graded according to the American Thoracic Society/Centers for Disease Control and Prevention guidelines [17]. Specimens in which the AFB smear results were categorized as grades 1 to 4 were defined as smear-positive. All clinical specimens were cultured on both solid and liquid medium for 6 weeks. To this end, decontaminated samples were inoculated into a mycobacterial growth indicator tube (MGIT 960 system; Becton Dickinson, Sparks, MD) and also onto 3% Ogawa agar (Shinyang, Seoul, Korea). All positive cultures were subjected to an AFB smear to confirm the presence of AFB and to exclude contamination. The Cobas TaqMan MTB test (Cobas MTB test) (Roche Diagnostics, Basel, Switzerland), which is a real-time PCR assay, was used to analyze sputum and pleural effusion samples [18].

### Statistical analysis

Data are presented as median and interquartile range (IQR) for continuous variables and as frequency (percentage) for categorical variables. Data were compared using the Mann-Whitney U test for continuous variables because of non-normality and with Pearson’s *chi*-square test or Fisher’s exact test for categorical variables. When the expected value in any cell of a contingency table was below five, Fisher’s exact test was used instead of Pearson’s *chi*-square test. Because lymphocyte-predominant pleural TB involved probable cases, the diagnostic yield of microbiological tests might be underestimated in lymphocyte-predominant pleural effusions. Therefore, we also compared microbiological characteristics between lymphocyte- and neutrophil-predominant pleural TB in the subgroup with a definite diagnosis. All tests were two-sided, and a *P*-value < 0.05 was considered significant. Data were analyzed using IBM SPSS Statistics for Windows, version 23.0 (Armonk, NY, USA).

## Results

### Patient characteristics

Of a total of 233 patients who met the criteria for pleural TB, 155 (66.5%) had a definite diagnosis and 78 (33.5%) had a probable diagnosis. Pleural biopsies were performed in six (2.6%) patients. We excluded 33 patients with pleural TB in which other cells predominated and which was not compatible with the definition of lymphocyte- or neutrophil-predominance; the characteristics of these patients are presented for reference purposes (Tables A and B in S1 File). In total, 200 patients were included in our study: 181 patients (90.5%) with lymphocyte-predominant pleural effusions and 19 (9.5%) with neutrophil-predominant effusions (Fig 1). One patient with neutrophil-predominant pleural effusion was included in the probable pleural TB group, since results of repeated pleural fluid analysis met the criteria for probable pleural TB despite negative microbiological tests.

Baseline and clinical characteristics of 200 patients, grouped according to the predominant cell type in pleural fluid, are shown in Table 1. Although there were no significant differences in presenting symptoms between the two groups, inflammatory serum markers, such as white blood cell (WBC) count (*P* = 0.001) and C-reactive protein (CRP) (*P* = 0.001), were higher with neutrophil-predominant pleural TB than with lymphocyte-predominant pleural TB. A chest CT was performed in 173 patients (86.5%) during the diagnostic workup, and there were no significant differences in pulmonary parenchymal lesions indicative of TB, on chest CT or X-ray, between the two groups.

**Table 1:** Baseline Characteristics of 200 Patients with Pleural TB.

	Total (n = 200) number (%), median (IQR)	Lymphocyte (n = 181) number (%), median (IQR)	Neutrophil (n = 19) number (%), median (IQR)	*P-value*
Demographics				
Male sex	126 (63.0)	116 (64.1)	10 (52.6)	0.454
Age, years	54.0 (37.2–71.5)	53.8 (37.5–71.8)	60.0 (29.7–68.6)	0.654
BMI, kg/m^2^	21.2 (18.6–23.6)	21.3 (18.7–23.7)	19.0 (18.3–22.3)	0.096
Comorbidity				
Diabetes mellitus	30 (15.0)	28 (15.5)	2 (10.5)	0.744
Chronic kidney disease	3 (1.5)	3 (1.7)	0	1.000
Cancer	8 (4.0)	7 (3.9)	1 (5.3)	0.557
Hematologic disease	2 (1.0)	2 (1.1)	0	1.000
Rheumatologic disease	1 (0.5)	1 (0.6)	0	1.000
Immunologic therapy	3 (1.5)	2 (1.1)	1 (5.3)	0.260
Clinical				
Fever (> 38°C)	69 (34.5)	60 (33.1)	9 (47.4)	0.310
Cough	115 (57.5)	104 (57.5)	11 (57.9)	1.000
Sputum	59 (29.5)	50 (27.6)	9 (47.4)	0.109
Pleuritic chest pain	82 (41.0)	75 (41.4)	7 (36.8)	0.809
Radiology^a^				
Lung parenchymal lesions suspicious of TB	121 (60.5)	106 (58.6)	15 (78.9)	0.091
Blood test				
WBC (/μl)	6,165 (5,095–7,628)	6,040 (5,085–7,170)	8,520 (6,490–10,370)	0.001
CRP (mg/dl)	4.9 (2.3–8.3)	4.5 (2.2–7.3)	8.6 (6.4–12.8)	0.001
Albumin	3.8 (3.4–4.1)	3.7 (3.4–4.1)	3.8 (2.8–4.1)	0.612

IQR, interquartile range; TB, tuberculosis; BMI, body mass index; CT, computed tomography; WBC, white blood cell; CRP, C-reactive protein

^a^ Radiologic findings were evaluated by chest CT or chest X-ray.

### Pleural fluid analysis and microbiological characteristics of pleural TB

The results of pleural fluid analysis for lymphocyte- and neutrophil-predominant pleural TB are compared in Table 2. Neutrophil-predominant pleural effusions had higher lactate dehydrogenase (LDH) (*P* < 0.001) and lower glucose (*P* = 0.022) levels than lymphocyte-predominant effusions. Compared to the normal range for healthy adults, the ADA level was elevated in both types of pleural TB; however, there was no significant difference between the two groups. Table 3 shows the microbiological differences between lymphocyte- and neutrophil-predominant pleural TB. Sputum AFB staining, MTB culture, and TB-PCR were performed in 155 patients (77%). With sputum samples, neutrophil-predominant pleural TB demonstrated a statistically significant, higher positive rate than did lymphocyte-predominant TB for both AFB stain (*P* = 0.003) and MTB culture (*P* = 0.023). In pleural fluids, neutrophil-predominant patients also had significantly higher diagnostic yields for MTB cultures (*P* < 0.001) and a higher TB-PCR positive rate (*P* = 0.001) compared to lymphocyte-predominant patients.

**Table 2:** Pleural Fluid Analysis of Lymphocyte- and Neutrophil-Predominant Pleural TB.

Data	Lymphocyte (n = 181) median (IQR)	Neutrophil (n = 19) median (IQR)	*P-value*
pH	7.3 (7.2–7.3)	7.3 (7.2–7.3)	0.830
Lymphocytes (%)	74 (65–85)	15 (5–29)	< 0.001
Neutrophils (%)	4 (1–12)	67 (55–77)	< 0.001
WBC (/μL)	2,480 (1,000–4,015)	1,780 (940–7,460)	0.905
ADA (IU/L)	88.5 (72.0–107.2)	81.1 (59.1–111)	0.496
LDH (U/L)	653 (462–944)	1,402 (858–1,917)	< 0.001
Protein (mg/dL)	4.99 (4.58–5.29)	5.10 (4.26–5.47)	0.986
Glucose (mg/dL)	91 (72–111)	77 (41–98)	0.022

IQR, interquartile range; TB, tuberculosis; WBC, white blood cell; ADA, adenosine deaminase; LDH, lactate dehydrogenase.

**Table 3:** Microbiological Characteristics of Lymphocyte- and Neutrophil-Predominant Pleural TB.

	Total (n = 200) number (%)	Lymphocyte (n = 181) number (%)	Neutrophil (n = 19) number (%)	*P-value*
Sputum (n = 155)				
AFB stain	24 (12.0)	17 (9.4)	7 (36.8)	0.003
Culture	67 (33.5)	56 (30.9)	11 (57.9)	0.023
TB-PCR	19 (9.5)	15 (8.3)	4 (21.1)	0.089
Pleural effusion (n = 200)				
AFB stain	5 (2.5)	0	5 (26.3)	< 0.001
Culture	56 (28.0)	41 (22.7)	15 (78.9)	< 0.001
TB-PCR	15 (7.5)	9 (5.0)	6 (31.6)	0.001

TB, tuberculosis; AFB, acid-fast bacilli; PCR, polymerase chain reaction.

### Microbiological characteristics of definite pleural TB

In subgroup analysis of patients with definite pleural TB, there were 108 patients with lymphocyte predominance and 18 with neutrophil predominance (Table 4). Neutrophil-predominant showed higher positivity than lymphocyte-predominant TB with the sputum AFB stain (*P* = 0.022). In pleural fluids, the diagnostic yields of MTB culture (*P* = 0.001) and the TB-PCR positive rate (*P* = 0.005) were significantly higher in neutrophil-predominant than in lymphocyte-predominant pleural effusions.

**Table 4:** Microbiological Characteristics of Lymphocyte- and Neutrophil-Predominant Definite Pleural TB.

	Total (n = 126) number (%)	Lymphocyte (n = 108) number (%)	Neutrophil (n = 18) number (%)	*P-value*
Sputum				
AFB stain	23 (18.3)	16 (14.8)	7 (38.9)	0.022
Culture	63 (50)	52 (48.1)	11 (61.1)	0.446
TB-PCR	19 (15.1)	15 (13.9)	4 (22.2)	0.474
Pleural effusion				
AFB stain	5 (4)	0	5 (27.8)	< 0.001
Culture	56 (44.4)	41 (38)	15 (83.8)	0.001
TB-PCR	14 (11.1)	8 (7.4)	6 (33.3)	0.005

TB, tuberculosis; AFB, acid-fast bacilli; PCR, polymerase chain reaction.

### Consecutive pleural fluid analysis in neutrophil-predominant pleural TB

Two consecutive, pleural fluid analyses were performed for seven of 19 patients with neutrophil-predominant pleural TB (Table 5), with a median time span of three days (IQR, 2–9 days) between the consecutive analyses. Four of seven patients revealed persistent neutrophil-predominant features in repeated pleural fluid analyses, although a shift towards an increasing ratio of lymphocytes over neutrophils was observed in three patients.

**Table 5:** Consecutive Pleural Fluid Analysis of Patients with Neutrophil-Predominant Pleural TB.

Patient No.	1st analysis			2nd analysis			Time span, days
	Lymphocytes (%)	Neutrophils (%)	ADA (IU/L)	Lymphocytes (%)	Neutrophils (%)	ADA (IU/L)	
1	36	55	99.2	38	29	99.7	1
2	14	78	105.5	12	80	10.7	4
3	5	85	111	22	70	115.1	9
4	15	77	81.1	3	95	36	11
5	20	55	69.3	41	20	59	2
6	14	76	69.3	43	21	66.5	3
7	0	93	206.6	0	95	186.5	2

TB, tuberculosis; ADA, adenosine deaminase.

## Discussion

The differentiation of pleural TB from parapneumonic effusion is generally determined by pleural fluid analysis demonstrating a lymphocyte-predominant exudate with high ADA versus a neutrophil-predominant exudate. However, recent studies reported an increased rate of neutrophil-predominant pleural TB [11, 12], and our study also found that 9.5% of patients with pleural TB had neutrophil predominance. We performed more detailed analysis of neutrophil-predominant pleural TB and showed that this form of pleural TB exhibits higher WBC counts and CRP in the blood, as well as higher LDH and lower glucose in the pleural fluid, than does lymphocyte-predominant pleural TB. This suggests a high-intensity inflammation response in the systemic circulation and pleural space in neutrophil-predominant pleural TB. Despite this increased inflammatory response, clinical manifestations, such as fever, cough, sputum or pleuritic pain, and radiological lung parenchymal abnormalities, did not differ between lymphocyte- and neutrophil-predominant pleural TB. These findings might be due to the higher sensitivity of assays for inflammatory markers than assessment of presented clinical manifestations.

Microbiologically, our study showed that neutrophil-predominant pleural TB had a higher yield of MTB in sputum and pleural fluid culture than lymphocyte-predominant pleural TB, which is consistent with a previous study [11]. Even when analysis was restricted to patients with a definite diagnosis to avoid the potentially diluting effect of probable cases of lymphocyte-predominant pleural TB, there was still a significant difference in microbiological characteristics between lymphocyte- and neutrophil-predominant pleural TB. In addition, despite similar concomitant lung parenchymal lesions, neutrophil-predominant pleural TB had a higher rate of AFB-positive sputum staining than the lymphocyte-predominant form, suggesting high infectivity of neutrophil-predominant pleural TB. These findings imply that microbiological tests should be conducted in neutrophil-predominant pleural effusion and that more precautions should be taken to prevent TB transmission by this group.

The introduction of liquid culture media has increased MTB detection rates [12, 19, 20], whereas previous studies that used only solid culture media reported a low positive rate of AFB staining [9] and MTB culture in pleural fluid [21, 22]. In the present study, the use of liquid media enabled the detection of one additional case (6.3%) from sputum and three additional cases (15.8%) from pleural effusion for neutrophil-predominant pleural TB, and 10 additional cases (7.2%) from sputum and 18 additional cases (9.9%) from pleural effusion for lymphocyte-predominant pleural TB (Fig A in S1 File). As the value of microbiological testing for the diagnosis of pleural TB is increasing through the use of liquid media [12, 19], clinicians should give more attention to microbiological approaches for diagnosing pleural TB, especially with neutrophil-predominant pleural TB.

Approximately 60% of patients with pleural TB had concomitant lung parenchymal abnormalities, which was consistent with a previous report showing that more than 80% of patients with pleural TB had pulmonary parenchymal abnormalities [13–15]. In addition, previous studies showed that patients with pleural TB often developed active pulmonary TB at a later time [23] and that a delay in anti-TB treatment of more than 14 days was associated with a worse clinical outcome after one year of follow-up in patients with neutrophil-predominant pleural TB [24]. Hence, clinicians should consider the possibility of pleural TB when they encounter neutrophil-predominant pleural effusion, especially in societies with a high TB incidence.

The traditional concept of pleural TB pathogenesis considers lymphocyte- and neutrophil-predominant features as sequential phases in the development of pleural TB. In this model, a rapid neutrophilic inflammatory response within the pleura in the early stage of pleural TB is followed by a prolonged, lymphocyte-driven, immune reaction and pleural granuloma formation [8]. However, our data showed that more than 50% of patients (n = 4 out of 7) with repeated pleural fluid analyses exhibited persistently neutrophil-predominant effusions. A previous study also reported that not all patients had converted to lymphocyte-predominant effusions during repeated pleural fluid analyses [11]. However, given the lack of accurate data on the time spans between initiation of symptoms (e.g., chest pain) and the first thoracentesis, it is not certain whether neutrophil-predominant pleural TB is a form of the disease distinct from lymphocyte-predominant pleural TB or represents the early stage in the sequelae of pleural TB. Because repeated thoracentesis and serial pleural fluid analyses were not conducted in all cases, prospective studies with serial pleural effusion evaluations are needed to explain the phenomenon of persistent neutrophil predominance.

Our study has several limitations. First, given the observational nature of the study, there is the possibility that selection bias or confounding factors may have influenced our findings. In addition, accurate data on the time spans between initiation of symptoms and thoracentesis were not available. Finally, our study subjects were collected from a single referral center. More studies are needed to confirm whether our study findings can be applied to all pleural TB cases.

## Conclusion

Neutrophil-predominant pleural TB demonstrates a more intense degree of inflammation and a higher positive rate of microbiological examinations compared to lymphocyte-predominant pleural TB. The possibility of pleural TB should be considered in neutrophil-predominant pleural effusions, and microbiological tests are necessary for the appropriate diagnosis of pleural TB.

## Supporting Information

S1 File Table A. Baseline Characteristics of 33 Patients with Non-lymphocyte-, Non-neutrophil-predominant Pleural TB. Table B. Pleural Fluid Analysis and Microbiological Characteristics of 33 Patients with Non-lymphocyte-, Non-neutrophil-predominant Pleural TB. Fig A. Diagnostic yields of cultures from (A) sputa and (B) pleural effusions. Y-axis indicates percentage of patients with lymphocyte-predominant (black bars) and neutrophil-predominant (hatched bars) TB pleurisy for each category of culture results. X-axis indicates (+) positive MTB growth in liquid and/or solid media, or (-) no growth detected.
(DOCX)

## Abbreviations

TB: tuberculosis
MTB: *Mycobacterium tuberculosis*
ADA: adenosine deaminase
AFB: acid-fast bacilli
PCR: polymerase chain reaction
CT: computed tomography
IQR: interquartile range
LDH: lactate dehydrogenase
