# Supplementary material for: Clinical and Laboratory Differences between Lymphocyte- and Neutrophil-Predominant Pleural Tuberculosis
Source: PLoS One. 2016 Oct 27;11(10):e0165428. doi: 10.1371/journal.pone.0165428 (PMC5082823; doi:10.1371/journal.pone.0165428)

**Supporting Information**

**Table A. Baseline Characteristics of 33 Patients with Non-lymphocyte-, Non-neutrophil-predominant Pleural TB.**

|  | Total (n = 33)  number (%), median (IQR) |
| --- | --- |
| Demographics |  |
| Male sex | 28 (84.8) |
| Age, years | 56.8 (28.9-71.3) |
| BMI, kg/m^2^ | 21.6 (19.9-23.6) |
| Comorbidity |  |
| Diabetes mellitus | 2 (6.1) |
| Chronic kidney disease | 2 (6.1) |
| Cancer | 3 (9.1) |
| Hematologic disease | 0 |
| Rheumatologic disease | 0 |
| Immunologic therapy | 0 |
| Clinical |  |
| Fever (> 38 ˚C) | 14 (42.4) |
| Cough | 20 (60.6) |
| Sputum | 13 (39.4) |
| Pleuritic chest pain | 11 (33.3) |
| Radiology^a^ |  |
| Lung parenchymal lesions | 21 (63.6) |
| suspicious of TB |  |
| Blood test |  |
| WBC (/µl) | 6,360 (5,050-8,205) |
| CRP (mg/dl) | 5.8 (2.5-9.7) |
| Albumin | 3.8 (3.0-4.1) |

IQR, interquartile range; TB, tuberculosis; BMI, body mass index; CT, computed tomography; WBC, white blood cell; CRP, C-reactive protein

^a^ Radiologic findings were evaluated by chest CT or chest X-ray.

**Table B. Pleural Fluid Analysis and Microbiological Characteristics of 33 Patients with Non-lymphocyte-, Non-neutrophil-predominant Pleural TB.**

| Data | Total (n = 33)  median (IQR) |
| --- | --- |
| **Pleural Fluid Analysis** |  |
| pH | 7.3 (7.2-7.3) |
| Lymphocytes (%) | 38 (30-43.5) |
| Neutrophils (%) | 19 (8-34) |
| Other cells (%) | 42 (30-58.5) |
| WBC (/µL) | 2,670 (1,000-5,805) |
| ADA (IU/L) | 84.5 (55.1-97.9) |
| LDH (U/L) | 748 (613.5-1019.5) |
| Protein (mg/dL) | 4.5 (4.1-5.2) |
| Glucose (mg/dL) | 99 (83.5-122.5) |
| **Microbiological Characteristics** |  |
| Sputum |  |
| AFB stain | 8 (24.2) |
| Culture | 16 (48.5) |
| TB-PCR | 4 (12.1) |
| Pleural effusion |  |
| AFB stain | 1 (3.0) |
| Culture | 15 (45.5) |
| TB-PCR | 6 (18.2) |

IQR, interquartile range; TB, tuberculosis; WBC, white blood cell; ADA, adenosine deaminase; LDH, lactate dehydrogenase; AFB, acid-fast bacilli; PCR, polymerase chain reaction

**Fig A.** Diagnostic yields of cultures from (A) sputa and (B) pleural effusions. Y-axis indicates percentage of patients with lymphocyte-predominant (black bars) and neutrophil-predominant (hatched bars) TB pleurisy for each category of culture results. X-axis indicates (+) positive MTB growth in liquid and/or solid media, or (-) no growth detected.


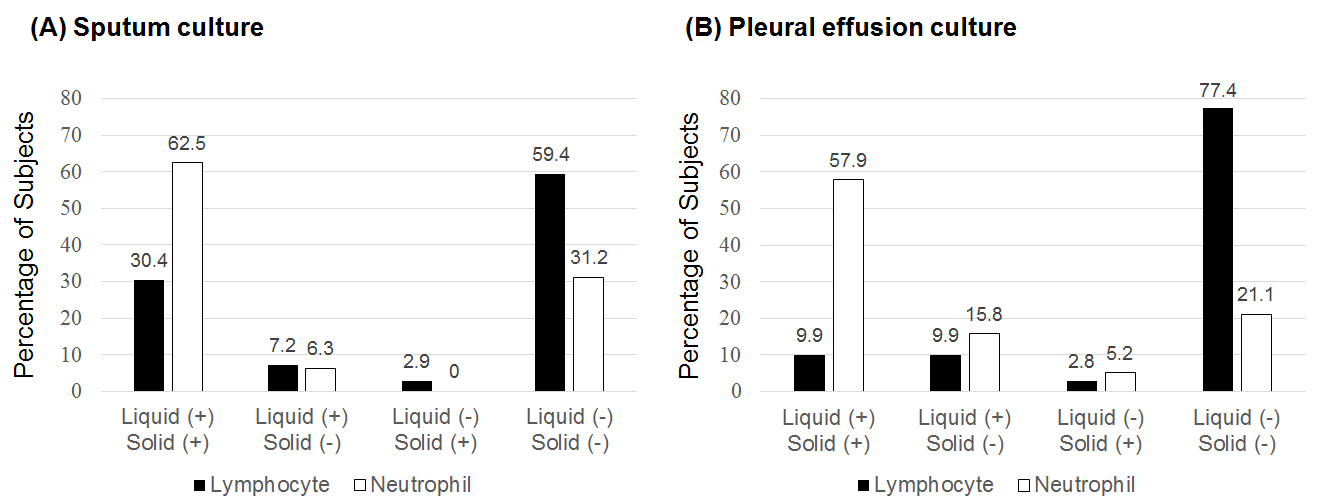

Supplement: S1 File — Table A. Baseline Characteristics of 33 Patients with Non-lymphocyte-, Non-neutrophil-predominant Pleural TB. Table B. Pleural Fluid Analysis and Microbiological Characteristics of 33 Patients with Non-lymphocyte-, Non-neutrophil-predominant Pleural TB. Fig A. Diagnostic yields of cultures from (A) sputa and (B) pleural effusions. Y-axis indicates percentage of patients with lymphocyte-predominant (black bars) and neutrophil-predominant (hatched bars) TB pleurisy for each category of culture results. X-axis indicates (+) positive MTB growth in liquid and/or solid media, or (-) no growth detected. (DOCX) [file pone.0165428.s001.docx]
